# Citrin Deficiency: Clinical and Nutritional Features

**DOI:** 10.3390/nu15102284

**Published:** 2023-05-12

**Authors:** Michiharu Komatsu, Naoki Tanaka, Takefumi Kimura, Masahide Yazaki

**Affiliations:** 1 Department of Gastroenterology, Suwa Red Cross Hospital, Suwa 392-8510, Nagano, Japan; 2 Department of Global Medical Research Promotion, Shinshu University Graduate School of Medicine, Matsumoto 390-8621, Nagano, Japan; 3 International Relations Office, Shinshu University School of Medicine, Matsumoto 390-8621, Nagano, Japan; 4 Research Center for Social Systems, Shinshu University, Matsumoto 390-8621, Nagano, Japan; 5 Department of Gastroenterology, Shinshu University School of Medicine, Matsumoto 390-8621, Nagano, Japan; 6 Department of Neuro-Health Innovation, Institute for Biomedical Sciences, Shinshu University, Matsumoto 390-8621, Nagano, Japan

**Keywords:** lean NAFLD, peculiar dietary habits, PPARα, avoidance of glycerol administration

## Abstract

*SLC25A13* gene mutations are responsible for diseases related to citrin deficiency (CD), such as neonatal intrahepatic cholestasis caused by citrin deficiency and adult-onset type II citrullinemia (CTLN2). From childhood to adulthood, CD patients are apparently healthy due to metabolic compensation with peculiar dietary habits—disliking high-carbohydrate foods and liking fat and protein-rich foods. Carbohydrate overload and alcohol consumption may trigger the sudden onset of CTLN2, inducing hyperammonemia and consciousness disturbance. Well-compensated asymptomatic CD patients are sometimes diagnosed as having non-obese (lean) non-alcoholic fatty liver disease and steatohepatitis, which have the risk of developing into liver cirrhosis and hepatocellular carcinoma. CD-induced fatty liver demonstrates significant suppression of peroxisome proliferator-activated receptor α and its downstream enzymes/proteins involved in fatty acid transport and oxidation and triglyceride secretion as a very low-density lipoprotein. Nutritional therapy is an essential and important treatment of CD, and medium-chain triglycerides oil and sodium pyruvate are useful for preventing hyperammonemia. We need to avoid the use of glycerol for treating brain edema by hyperammonemia. This review summarizes the clinical and nutritional features of CD-associated fatty liver disease and promising nutritional interventions.

## 1. Introduction

Citrin, a liver-type of aralar, is associated with the energy supply in hepatocytes through an aspartate–glutamate carrier (AGC). The AGC plays an important role in replacing mitochondrial aspartate with cytosolic glutamate and a proton [1,2,3]. This process is mandatory to shift cytosolic nicotinamide adenine dinucleotide (NADH) into the mitochondria and to balance the mitochondrial and cytosolic NADH-NAD+ concentrations through the malate–aspartate shuttle. NADH is necessary for producing adenosine triphosphate (ATP) through the oxidative phosphorylation pathway. Aspartate is converted into argininosuccinate by argininosuccinate synthetase (ASS) in the cytosol. Arginosuccinate is utilized as a substrate of the urea cycle. Citrin is involved with neutralizing ammonia by the urea cycle. Thus, citrin deficiency (CD) impairs the urea cycle, leading to hyperammonemia [2,3].

Citrin is encoded by the *SLC25A13* gene, which is located on chromosome 7q21.3 [2,3,4]. CD is an autosomal recessive metabolic disease caused by a mutation of the *SLC25A13* gene. The first case of CD was reported in Japan, and the relatively common mutations were identified in East Asia and Southeast Asia [4,5,6,7]. The prevalence of these mutations has been reported to be 1/65 in China, 1/112 in Korea, and 1/69 in Japan [3,8,9]. The overall mutation carrier rates among newborns could be as high as 2% (107/5332) in southern China [10]. The mutation carrier rate is 1/51 in Guangdong Province, with an estimated CD prevalence of 1/10,053, whereas a lower carrier frequency (1/95) is observed in Shaanxi Province, with a theoretical incidence of 1/35,865 [11], showing a big regional difference in the frequency of mutation even in China. Recently, these mutations have been reported in various races and many non-Asian countries, such as the UK, France, and Canada [12,13,14], so CD is recognized as a pan-ethnic global disease.

CD patients demonstrate some typical clinical features with age, i.e., neonatal intrahepatic cholestasis caused by citrin deficiency (NICCD) in neonates and infants, an asymptomatic stage due to adaptation/compensation in children and young adults, and adult-onset type II citrullinemia (CTLN2) [2,3,15]. The characteristics of NICCD are prolonged jaundice, intrahepatic cholestasis, low body weight, fatty liver, hypoproteinemia, hypoglycemia, coagulopathy, and citrullinemia, and usually resolve by 1 year of age. A few patients progress into liver failure requiring liver transplantation [16,17]. After the neonate and infant phases, many NICCD patients are considered to be asymptomatic, except for their dietary preferences [18]. However, recent studies have shown that CD patients show several clinical symptoms, such as fatigue, dyslipidemia, and digestive disorders, which are designated as failure to thrive and dyslipidemia caused by CD (FTTDCD). The common symptoms of FTTDCD are hypoglycemia, lethargy, hypertriglyceridemia, pancreatitis, and non-alcoholic fatty liver disease (NAFLD) and steatohepatitis (NASH) unrelated to obesity and metabolic syndrome [18]. Recently, some reports have described even more cases developing into liver cirrhosis and liver cancer [19,20,21,22,23]. When clinicians encounter a young patient suffering from recurrent pancreatitis or lean NAFLD, the possibility of CD should be considered, and the patient’s food habits should be determined. Peculiar food habits, such as disliking carbohydrates and preferring fat and protein-rich foods, such as beans, cheese, and squid, are characteristic of CD. This dietary habit may be a consequence of needing to compensate for the metabolic disruption caused by CD. CTLN2 is caused by a sudden attack of hyperammonemia and various neuropsychological abnormalities, such as disorientation, abnormal behavior, convulsions, and coma in adolescents and adults (11–79 years). As a result, rapidly progressive and irreversible brain edema may lead to death. The prognosis of CTLN2 patients exhibiting hyperammonemia-induced encephalopathy is pitiable. If ammonia-lowering and neuroprotective therapies are ineffective, liver transplantation must be decided to save their lives. Therefore, early diagnosis and the appropriate treatment of CD, especially interventions to prevent hyperammonemia-induced encephalopathy, are essential for CD patients to improve their prognosis. Here, we describe the clinical characteristics of NAFLD/NASH with CD, especially the differences in the clinical features from conventional NAFLD/NASH associated with metabolic syndrome, and their mechanisms and recent nutritional interventions for CD patients.

## 2. Clinical Characteristics of Fatty Liver Due to CD

**Figure 1:**
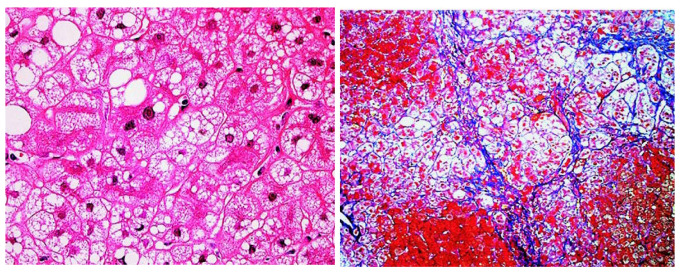
Liver histology of CD patient. Hematoxylin and eosin staining (**left**, ×100) revealed microvesicular and macrovesicular steatosis and ballooned hepatocytes. Azan–Mallory staining (**right**, ×40) showed marked pericellular fibrosis with disorganization of hepatic lobules. This figure is reprinted/adapted with permission from Ref. [24]. 2007 by the AGA Institute.

CD has been demonstrated to present with hepatic steatosis and steatohepatitis [23]. We experienced a case of a patient with NAFLD who was later diagnosed as having an *SLC25A13* gene mutation [24]. His body weight was 50 kg with a body mass index (BMI) of 17.3 kg/m^2^, but his blood examination showed elevated serum aminotransferase levels and he had hepatic steatosis by abdominal ultrasonography, leading to the clinical diagnosis of non-obese (lean) NAFLD. A liver biopsy revealed macrovesicular steatosis, ballooned hepatocytes, and apparent pericellular fibrosis, which was diagnosed as NASH (Figure 1). Later, he drank alcohol accidentally, and hyperammonemia and serious encephalopathy emerged suddenly. The patient was diagnosed as having CTLN2, but medical treatments had no effect to recover his consciousness level. So, he received liver transplantation [25]. This case suggests the necessity of realizing the clinical feature and course of CD before the onset of neurological symptoms.

**Figure 2:**
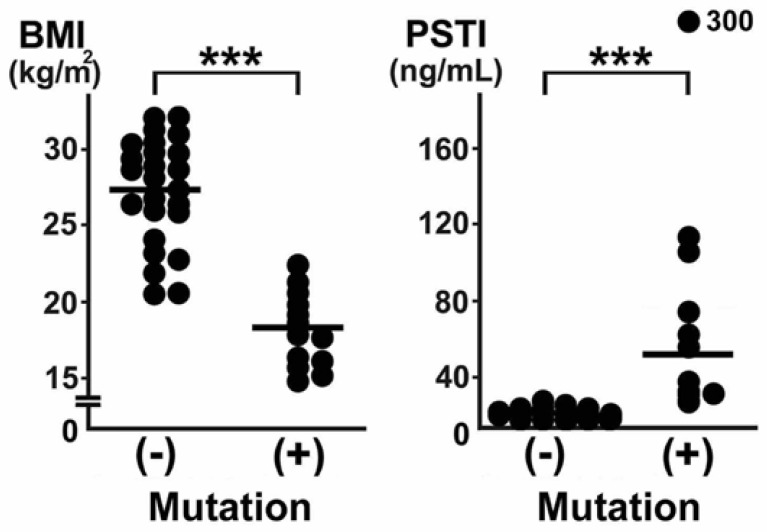
Comparison of BMI and serum PSTI between fatty liver patients without (−) and with (+) *SLC25A13* gene mutations. (−), conventional NAFLD/NASH; (+), CD-associated fatty liver disease. Each value is plotted, and median values are indicated in the lines. The cut-off values of BMI and serum PSTI were calculated as 20 kg/m^2^ and 29 ng/mL, respectively. ***, *p* < 0.001. This figure is reprinted/adapted with permission from Ref. [26]. 2008 European Association for the Study of the Liver. Published by Elsevier Ireland Ltd. All rights reserved.

We summarized the clinical characteristics of 19 CTLN2 patients compared with 25 fatty liver patients without *SLC25A13* gene mutation [26]. In the CD patients, six (32%) had notably elevated serum triglycerides (TGs). Surprisingly, even in the asymptomatic phase, hypertriglyceridemia, fatty liver, and the elevation of serum alanine aminotransferase (ALT) levels had been detected in three (16%), five (26%), and eight (42%) patients, respectively. A past history of pancreatitis was recognized in five (26%) patients before the diagnosis of CTLN2. No CTLN2 patients had drunk alcohol. One patient had a past history of prolonged neonatal jaundice. At the time of admission, elevated serum ALT and γ-glutamyltransferase levels and fatty liver were observed in 17 (89%), 15 (79%), and 17 (89%) patients, respectively. The median BMI values were as low as 18.3 kg/m^2^ in the CTLN2 patients with fatty liver, and all patients were lean (Figure 2). Moreover, the CTLN2 patients had a tendency to have a decrease in their waist circumference. These findings indicate the possibility that asymptomatic CD patients are involved in cases of non-obese/lean NAFLD/NASH.

Liver histological findings of the CD patients revealed macrovesicular and microvesicular steatosis, in addition to lobular/portal inflammation and pericellular fibrosis (Figure 1), and some cases met the criteria of NASH. The clinical characteristics of NAFLD with CD were a low BMI, no association with diabetes, liver dysfunction, high serum pancreatic secretory trypsin inhibitor (PSTI) concentrations, and a past history of pancreatitis (Figure 2). PSTI is known as serine protease inhibitor Kazal type 1 (SPINK1) and reduces damage in pancreatic acinar cells. *PSTI/SPINK1* gene expression is associated with the onset of pancreatitis [27]. Metabolic disorder with CD leads to continuously injured pancreatic acinar cells, and serum PSTI levels are elevated. Pancreatitis is an interesting characteristic to recognize CD features [28]. Although pancreatitis is associated with heavy drinking, bile duct stones, and some drugs, pancreatitis in CD patients occurs without any incitement. Indeed, pancreatic exocrine insufficiency is sometimes underlying non-obese NAFLD/NASH [29]. The CTLN2 patients had peculiar dietary habits to avoid carbohydrate-rich foods and prefer high-fat high-protein foods, such as beans, eggs, squid, or cheese, presumably due to metabolic adaptation. Therefore, when clinicians encounter non-obese NAFLD/NASH patients, we recommend interviewing them about their food preferences and past history of pancreatitis, prolonged neonatal jaundice, and hypertriglyceridemia and to measure their serum PSTI, considering the possibility of CD in the absence of neurological symptoms.

## 3. Possible Mechanism of Steatogenesis Due to CD

**Figure 3:**
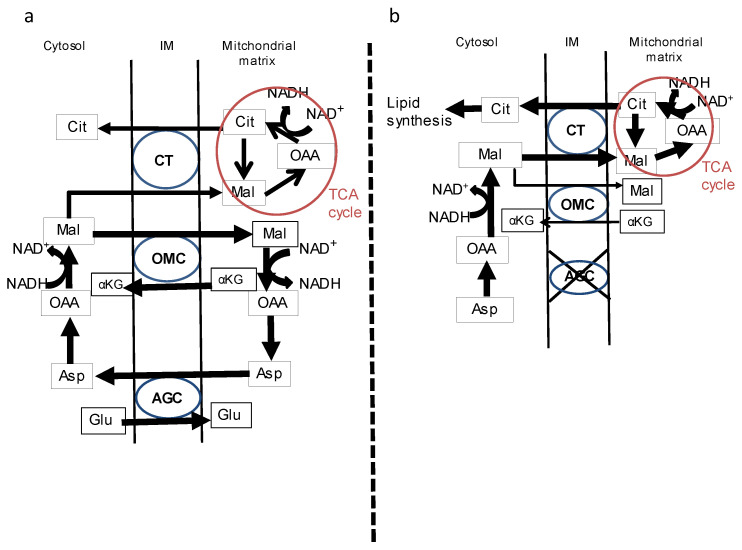
A schematic diagram of metabolic change in CD patients. (**a**) Citrin (aspartate–glutamate carrier; AGC) works as part of the malate–aspartate shuttle and this shuttle is the main pathway to translocate NADH from the cytosol to the mitochondrial matrix. The NADH-NAD+ balance is kept in good condition in the cytosol and mitochondrial matrix. (**b**) Since citrin (AGC) does not work sufficiently in CD patients’ hepatocytes, the NADH supply depends on the TCA cycle. As a result, citrate is increased in the cytosol and used as a source of fatty acid synthesis. Cit, citrate; OAA, oxaloacetate; Mal, malate; αKG, α-ketoglutarate; Asp, aspartate; Glu, glutamate; NADH, nicotinamide adenine dinucleotide; CT, citrate transporter; OMC, oxoglutarate malate transporter; IM, intermembrane space. This figure is reprinted/adapted with permission from Ref. [26]. 2008 European Association for the Study of the Liver. Published by Elsevier Ireland Ltd.

Glucose is metabolized to pyruvate through the glycolytic pathway, and pyruvate transfers in the tricarboxylic acid (TCA) cycle into mitochondria. Pyruvate is decarboxylated to acetyl-coenzyme A (CoA) by pyruvate dehydrogenase and converted into citrate with the condensation reaction by citrate synthase. Citrate is transferred to the cytosol and is a compounded fatty acid (FA) and TG. The mechanism of steatogenesis in a CD liver can be explained as follows: The malate–aspartate shuttle is a critical metabolic pathway to supply NADH to the mitochondria in the hepatocyte. Malate is hydrolyzed by malate dehydrogenase, and then NAD+ and a proton are conjugated as NADH. This shuttle does not work effectively in CD patients’ livers, and a heavy load is applied to the malate–citrate shuttle to treat malate in the mitochondrial matrix and intermembrane space [30]. Consequently, the citrate becomes redundant in the cytosol and is used for lipid synthesis (Figure 3a,b). Finally, the citrate and FAs accumulate, and remodel the TGs, in the hepatocyte. It was reported that the elevated mRNA levels of ATP-citrate lyase were confirmed in the livers of citrin/mitochondrial glycerol-3-phosphate dehydrogenase double-knockout mouse, a suitable model of human CD. The mRNA levels of CD36 (FA translocase) were also elevated in these mouse livers [31]. It is expected that the ability of CD livers to uptake non-esterified FAs from plasma is exacerbated. According to these facts, the original defect of the malate–aspartate shuttle leads to changes in the lipid metabolism in CD patients’ hepatocytes, and fatty liver is formed to use a large number of FAs and TG in the cytosol. This is a luculent feature to explain the constitution of a fatty liver in CD patients.

**Figure 4:**
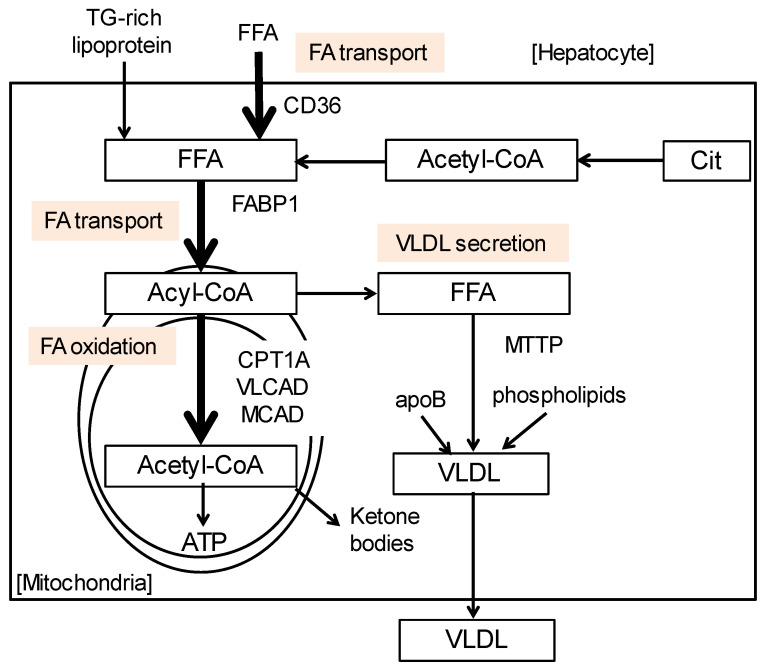
FA/TG metabolism in the liver. The mRNA-encoding enzymes/proteins associated with FA oxidation, very low-density lipoprotein secretion, and FA transport were markedly downregulated in CD livers. Cit, citrate; CPT1A, carnitine palmitoyl–CoA transferase 1α; FABP1, FA-binding protein 1; FFA, free fatty acid; MCAD, medium-chain acyl-CoA dehydrogenase; MTTP, microsomal TG transfer protein; VLCAD, very-long-chain acyl-CoA dehydrogenase; VLDL, very-low-density lipoprotein.

The plasma ketone body levels of CD patients are comparably lower than healthy individuals [32], suggesting that the activity to catabolize FAs in the mitochondria might be downregulated [33,34]. On the other hand, citrin is necessary for urea metabolism and carbohydrate metabolism. It is widely known that urea cycle disorder, such as carbamoyl-phosphate synthase deficiency and ornithine transcarbamoylase deficiency, shows hepatic steatosis, especially microvesicular steatosis [35]. We investigated the expressions of genes associated with TG and FA metabolism using CD patients with fatty liver compared with healthy individuals. To analyze the mechanism of steatogenesis in CD patients, the hepatic mRNA levels of genes associated with FA uptake, transport, and activation were determined (Figure 4). The mRNA-encoding enzymes/proteins associated with FA oxidation (carnitine palmitoyl-CoA transferase 1α, medium- and very-long-chain acyl-CoA dehydrogenases, and acyl-CoA oxidase 1), very-low-density lipoprotein secretion (microsomal TG transfer protein (MTTP)) and FA transport (CD36 and FA-binding protein 1) were markedly suppressed in CTLN2 patients’ livers. The decreases in these molecules were correlated with the degree of fatty liver. These enzymes/proteins are regulated by peroxisome proliferator-activated receptor α (PPARα), a master regulator of hepatic lipid metabolism [34,36,37]. Actually, the PPARα mRNA levels were significantly downregulated. The serum ketone bodies, indicators of mitochondrial β-oxidation activity, were also significantly decreased in CTLN2 patients and inversely correlated with the severity of steatosis [38]. According to these results, the mechanism of steatogenesis due to CTLN2 may be explained by decreased PPARα activity and the ensuing decline in the consumption of TG and FA in the mitochondria.

Urea cycle disorders are sometimes complicated by fatty liver, and oxidative stress has an important role in developing NAFLD and NASH [39]. Arginine is an important component in the urea cycle and needs to produce nitric oxygen by nitric oxide synthesis [40]. Because urea cycle disorder patients have difficulty securing sufficient arginine for a homeostasis-retaining composition in hepatocytes, reduced nitric oxygen levels lead to increased free radical production due to the uncoupling of nitric oxide synthesis [41,42]. Free radicals are known to result in liver inflammation and fibrosis in conventional NAFLD and NASH [43,44], which also occurs in fatty liver disease caused by urea cycle disorder.

## 4. Treatment for CD

One of the metabolic effects of CD is the inhibition of gluconeogenesis from lactate and glycerol in hepatocytes due to NADH-NAD+ imbalance. NADH needs to be supplied into mitochondria to generate ATP through malate-aspartate shuttle, so CD patients tend to lack enough energy supplementations. Protein and fat are used as the main source of energy in CD patients’ livers, and stable intakes of these are important in the nutritional approach for CD. Saeki et al. compared the food intake of 18 Japanese CD patients from 1 to 33 years in age to an age- and sex-matched general Japanese population. In the general Japanese population, the contribution ratios of energy from three major nutrients to the total energy intake were 54–58%, 25–30%, and 14–15% from carbohydrates, fats, and proteins, respectively. However, the ratios in CD patients were 37 ± 7%, 44 ± 5%, and 19 ± 2% from carbohydrates, fats, and proteins, respectively, indicating a preference for low-carbohydrate and high-fat high-protein foods [45]. This trend was similar to the NICCD and FTTDCD patients in the UK. The median percentages of energy contribution from carbohydrates, fats, and proteins were 42%, 40%, and 19% [46].

Okamoto et al. evaluated the preference of 435 food items in 70 Japanese CD patients and 55 age-matched control subjects. The foods marked as “dislike” were significantly greater in patients (37% vs. 16%). Specifically, the preference for vegetables, seaweed, fruits, confectioneries/beverages, potatoes, and grains was significantly lower in the patients [47]. Therefore, asking patients about not only their preference for beans, nuts, meats, and eggs but also clearly disliked foods may be useful to estimate the possibility of CD.

We show the summary of treatment for CD in Table 1. The most important therapy is to intervene in their diet to keep the proper protein–fat–carbohydrate ratio, for example, 15–25% protein, 40–50% fat, and 30–40% carbohydrate are recommended [18,47,48]. In addition to protein- and fat-rich diets, low-carbohydrate diets recover CD patients from general fatigue, gastrointestinal symptoms, and neurological abnormalities. On the contrary, correcting the peculiar food habits, i.e., a preference for fat- and protein-rich foods and an aversion to carbohydrates must be avoided for asymptomatic CD or FTTDCD patients due to the high risk of inducing hyperammonemia and neuropsychiatric symptoms [49]. This is quite important for clinical nutritionists to recognize the possibility of CD in patients with dyslipidemia, lean NAFLD/NASH, and pancreatitis of unknown etiology and the toxicity of carbohydrates for CD.

CD patients may show hypoglycemic symptoms during long-term fasting. Even in the presence of hypoglycemia, the patients should avoid sugar-rich foods and beverages and instead have high-fat high-protein foods, such as chocolate, cheesecake, and milk. We should pay careful attention to the unintentional excessive administration of carbohydrates to CD patients in schools and hospitals. When oral intake is difficult, a minimal glucose solution (~5% glucose concentration) should be given continuously and intravenously. It was reported that oral glucose tolerance tests of CD-suspected patients induced mood deterioration and hyperammonemia, and they recovered with MCT administration [50].

As stated in Section 2, forced drinking can trigger hyperammonemia and hepatic encephalopathy in neuropsychologically free CD patients with NAFLD, requiring liver transplantation [24]. Basically, CD patients dislike alcohol consumption even in small amounts because of the emergence of pre-CTLN2 symptoms. Careful attention should be paid to not induce a critical situation.

For the treatment of brain edema, administering a drip injection of glycerol is contraindicated for CD patients. Glycerol is used as an osmotic agent for the treatment of brain edema to reduce the brain water content. Glycerol is finally metabolized in the liver into pyruvate, but not glucose, leading to increased NADH generation and worsening hyperammonemia in patients with CD. Indeed, some cases have reported that CD patients given glycerol had aggravated encephalopathy and mostly died shortly after an infusion of glycerol [2,51,52]. Glycerol toxicity for CD patients was disclosed in a previous report using modified mouse models [31]. Therefore, clinicians should administer mannitol instead of glycerol for the treatment of brain edema due to CD because mannitol is barely metabolized in the body, and intravenous administration does not influence cytosolic NADH generation even in the CD state [51].

**Table 1:** Treatment for CD.

Treatment	Target Disease	Concept	References
Low-carbohydrate diet	NICCD CTLN2	Basal therapy	[18,47,48]
MCT + lactose-restricted formula	NICCD CTLN2	Energy supply Correction of metabolic disorder	[53,54,55,56,57,58]
Sodium pyruvate	CTLN2	Energy supply Correction of metabolic disorder	[59,60,61,62]
Ursodeoxycholic acid	NICCD	Hepatoprotection Anti-cholestasis	[18,63,64,65]
Mannitol	NICCD CTLN2	Osmotic agent for treating brain edema due to hyperammonemia	[51]
Liver transplantation *	NICCD CTLN2	Ultimate fundamental therapy	[16,66,67,68,69]

* In Japan, liver transplantation is performed on recipients under 65 years old. CTLN-2, adult-onset type II citrullinemia; NICCD, neonatal intrahepatic cholestasis caused by citrin deficiency; MCT, medium-chain triglycerides.

The usefulness of medium-chain TG (MCT) milk/oil was reported for improving CD patients’ conditions. MCT milk and lactose-restricted formula is an effective therapy for intrahepatic cholestasis in NICCD [53,54,55,56]. MCT can be absorbed from the intestine even in the presence of cholestasis because its absorption does not require the formation of micelle with bile acids. MCT is absorbed as medium-chain free FAs and easily metabolized to acetyl-CoA by β-oxidation in the mitochondria and increased ATP levels in hepatocytes. MCT has become a basic energy source in CD patients’ livers and has improved the levels of specific markers for CD, such as alkaline phosphatase, alpha-fetoprotein, and PSTI [53,54]. Moreover, it was documented that an MCT supplement with a low-carbohydrate formula had a favorable effect on hyperammonemic encephalopathy in CTLN2 patients [57,58].

The medication of sodium pyruvate is to supply a source for the TCA cycle to oxidize cytosolic NADH to NAD^+^ by lactate dehydrogenase reaction and to generate ATP in the liver [59,60]. In previous reports, after the patients have been given sodium pyruvate and had a low-carbohydrate meal, the frequency of attacks of encephalopathy markedly decreased [60,61]. However, this treatment neither improved the Fischer ratio (branched-chain amino acid/aromatic amino acid) or citrullinemia nor prevented the relapse of encephalopathy [57,62].

Ursodeoxycholic acid has been used in model mice and given to infants with intrahepatic cholestasis for NICCD [63,64,65], but we should not administer it in patients suffering from obstructive jaundice and acute liver failure [18]. Following treatment with the foregoing therapies, if consciousness disturbance and encephalopathy due to hyperammonemia are uncontrollable and recurring, liver transplantation is the most promising treatment for CD patients [16,66,67].

Liver transplantation is an established therapy for CTLN2 [67,68,69]. However, it imposes a severe burden on CD patients and donors, and CD patients cannot receive it as required medical care anytime. So, we have to make efforts to keep the proper nutritional adjustments for metabolic disorders and to prevent the onset of CTLN2.

Because of the abovementioned descriptions, it is possible that supplying energy to the liver might be a therapeutic approach for CD patients. According to our findings of the mechanisms of a fatty liver with CD, the constant upregulation of PPARα activity is thought to be a novel target for CD treatment. It leads to the amelioration of FA/TG accumulation and a stable supply of ATP in CD livers. An agent such as bezafibrate, an activator of PPARα, might be useful to treat fatty liver due to CD under the conditions of well-balanced metabolic situations in the liver [70].

## 5. Concluding Remarks: Suggestions for Clinicians and Dietitians

We reviewed the clinical characteristics of CD patients. CD patients mimic NAFLD and are apparently healthy individuals with unique food preferences before the onset of CTLN2. The most important point for such patients is that clinicians consider the possibility of CD in non-obese patients with fatty liver disease and prevent iatrogenic aggravation, such as the onset of hyperammonemia. In addition to peculiar dietary habits, a low BMI, high serum PSTI, and a past history of pancreatitis will be a clue to correct the diagnosis of CD.

Clinicians and dietarians need to pay attention to the dietary instructions for CD patients. The forced correction of peculiar dietary habits, i.e., a preference for a high-fat and high-protein diet and disliking carbohydrates, as well as forced consumption of carbohydrate-rich foods and sweet beverages/alcohol may cause various clinical symptoms, such as general fatigue, appetite loss, liver dysfunction, neurological abnormalities, hyperammonemia, and liver failure. We need to maintain their fat-rich and protein-rich diets, such as chocolate, cheese, and eggs, and supervise CD patients to avoid alcohol intake. Clinicians should avoid administering high-sugar solutions to CD patients, which may cause iatrogenic hyperammonemia, brain edema, and death.

## Data Availability

Not applicable.

## Abbreviations

AGC, aspartate–glutamate carrier; ALT, alanine aminotransferase; ASS, argininosuccinate synthetase; ATP, adenosine triphosphate; BMI, body mass index; CD: citrin deficiency; CoA, coenzyme A; CTLN2, adult-onset type II citrullinemia; FA, fatty acid; MCT, medium-chain triglycerides; MTTP, microsomal TG transfer protein; NADH, nicotinamide adenine dinucleotide; NAFLD, non-alcoholic fatty liver disease; NASH, non-alcoholic steatohepatitis; NICCD, neonatal intrahepatic cholestasis caused by citrin deficiency; PPARα, peroxisome proliferator-activated receptor α; PSTI, pancreatic secretory trypsin inhibitor; SPINK1, serine protease inhibitor Kazal type 1; TCA, tricarboxylic acid; TG, triglycerides.
